# Efficacy and safety of givosiran for acute hepatic porphyria: 24‐month interim analysis of the randomized phase 3 ENVISION study

**DOI:** 10.1111/liv.15090

**Published:** 2021-11-16

**Authors:** Paolo Ventura, Herbert L. Bonkovsky, Laurent Gouya, Paula Aguilera‐Peiró, D. Montgomery Bissell, Penelope E. Stein, Manisha Balwani, D. Karl E. Anderson, Charles Parker, David J. Kuter, Susana Monroy, Jeeyoung Oh, Bruce Ritchie, John J. Ko, Zhaowei Hua, Marianne T. Sweetser, Eliane Sardh, for the ENVISION Investigators

**Affiliations:** ^1^ Department of Surgical and Medical Sciences for Children and Adults, Internal Medicine Unit University of Modena and Reggio Emilia Modena Italy; ^2^ Section on Gastroenterology and Hepatology Wake Forest University/North Carolina Baptist Medical Center Winston‐Salem NC USA; ^3^ Centre Français des Porphyries Paris France; ^4^ Hospital Clinic Barcelona Barcelona Spain; ^5^ UCSF Liver Center and Porphyria Center, University of California San Francisco CA USA; ^6^ King’s College Hospital London UK; ^7^ Department of Genetics and Genomic Sciences, Icahn School of Medicine at Mount Sinai New York NY USA; ^8^ University of Texas Medical Branch Galveston TX USA; ^9^ University of Utah Salt Lake City UT USA; ^10^ Center for Hematology Massachusetts General Hospital Boston MA USA; ^11^ Instituto Nacional de Pediatría Mexico City Mexico; ^12^ Konkuk University Medical Center Seoul South Korea; ^13^ University of Alberta Hospital Edmonton Canada; ^14^ Alnylam Pharmaceuticals Cambridge MA USA; ^15^ Porphyria Centre Sweden, Centre for Inherited Metabolic Diseases, Karolinska Institutet, Karolinska University Hospital Stockholm Sweden

**Keywords:** Acute hepatic porphyria, ALA synthase‐1, givosiran, health‐related quality of life, RNAi therapeutics

## Abstract

**Background & Aims:**

Upregulation of hepatic delta‐aminolevulinic acid synthase 1 with accumulation of potentially toxic heme precursors delta‐aminolevulinic acid and porphobilinogen is fundamental to the pathogenesis of acute hepatic porphyria. Aims: evaluate long‐term efficacy and safety of givosiran in acute hepatic porphyria.

**Methods:**

Interim analysis of ongoing ENVISION study (NCT03338816), after all active patients completed their Month 24 visit. Patients with acute hepatic porphyria (≥12 years) with recurrent attacks received givosiran (2.5 mg/kg monthly) (n = 48) or placebo (n = 46) for 6 months (double‐blind period); 93 received givosiran (2.5 mg or 1.25 mg/kg monthly) in the open‐label extension (continuous givosiran, n = 47/48; placebo crossover, n = 46/46). Endpoints included annualized attack rate, urinary delta‐aminolevulinic acid and porphobilinogen levels, hemin use, daily worst pain, quality of life, and adverse events.

**Results:**

Patients receiving continuous givosiran had sustained annualized attack rate reduction (median 1.0 in double‐blind period, 0.0 in open‐label extension); in placebo crossover patients, median annualized attack rate decreased from 10.7 to 1.4. Median annualized days of hemin use were 0.0 (double‐blind period) and 0.0 (open‐label extension) for continuous givosiran patients and reduced from 14.98 to 0.71 for placebo crossover patients. Long‐term givosiran led to sustained lowering of delta‐aminolevulinic acid and porphobilinogen and improvements in daily worst pain and quality of life. Safety findings were consistent with the double‐blind period.

**Conclusions:**

Long‐term givosiran has an acceptable safety profile and significantly benefits acute hepatic porphyria patients with recurrent attacks by reducing attack frequency, hemin use, and severity of daily worst pain while improving quality of life.

## Graphical abstract

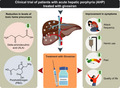

**Abbreviations**
AAR: annualized attack rate
AE: adverse event
AHP: acute hepatic porphyria
AIP: acute intermittent porphyria
ALA: delta‐aminolevulinic acid
ALAS1: delta‐aminolevulinic acid synthase 1
ALT: alanine aminotransferase
CKD: chronic kidney disease
Cr: creatinine
DB: double‐blind
eGFR: estimated glomerular filtration rate
EQ‐5D: EuroQOL‐5 Dimension
EQ‐VAS: EuroQol‐visual analog scale
EVGFP: Excellent, Very Good, Good, Fair, Poor
Givo: givosiran
HCP: hereditary coproporphyria
ISR: injection‐site reaction
IV: intravenous
MCS: Mental Component Summary
MedDRA: *M* *edical Dictionary for Regulatory Activities*
mRNA: messenger RNA
OLE: open‐label extension
PBG: porphobilinogen
PBO: placebo
PCS: Physical Component Summary
PGIC: Patient Global Impression of Change
PPEQ: Porphyria Patient Experience Questionnaire
QM: once monthly
QOL: quality of life
SAE: serious adverse event
SF‐12v2: 12‐Item Short Form Health Survey Version 2
SMQ: standardized MedDRA query
ULN: upper limit of normal
VP: variegate porphyria

**Lay Summary**

Acute hepatic porphyria is a rare genetic disease that involves potentially life‐threatening acute attacks and, for some patients, persistent symptoms impacting their ability to perform daily activities. In this evaluation of information compiled from the ongoing ENVISION study, long‐term givosiran treatment benefited acute hepatic porphyria patients with repeated attacks by reducing the number of attacks, hemin use, and daily pain while improving quality of life. Long‐term givosiran use is safe and effective for patients with acute hepatic porphyria who experience repeated attacks.

**Key points**

- After a 6‐month double‐blind period, 93 patients with acute hepatic porphyria and recurrent attacks received givosiran in the 30‐month open‐label extension period (continuous givosiran, n=47/48; placebo crossover, n=46/46); data from the 24‐month interim analysis are reported here.
- Continuous givosiran patients had sustained annualized attack rate reduction; in placebo crossover patients, median annualized attack rate decreased from 10.7 (double‐blind period) to 1.4 (open‐label extension period).
- Long‐term givosiran treatment led to sustained lowering of delta‐aminolevulinic acid and porphobilinogen and improvements in daily worst pain and patient‐reported assessments of quality of life.
- Long‐term givosiran treatment was well tolerated.

## 1 INTRODUCTION

Acute hepatic porphyria (AHP) is a family of four rare genetic diseases characterized by potentially life‐threatening acute attacks and, for some patients, chronic manifestations impacting daily functioning and quality of life (QOL).^1^, ^2^, ^3^, ^4^ The AHP types are acute intermittent porphyria (AIP; most common), variegate porphyria (VP), hereditary coproporphyria (HCP), and delta‐aminolevulinic acid (ALA) dehydratase–deficiency porphyria.^2^, ^5^ Clinical manifestations are due to pathogenic mutations leading to deficiency in an enzyme of hepatic heme biosynthesis.^6^ These defects predispose for triggering factors inducing delta‐aminolevulinic acid synthase 1 (ALAS1), the initial and normally rate‐controlling enzyme of the heme biosynthesis pathway^7^, ^8^; trigger factors may lead to further induction of ALAS1.^9^ In AHP, this can lead to accumulation of the potentially toxic porphyrin precursors ALA and porphobilinogen (PBG), thought to be causal for disease manifestations, as well as porphyrins.^9^, ^10^, ^11^

The most severe symptoms of AHP occur during acute neurovisceral attacks, which manifest most commonly as severe abdominal pain, nausea, vomiting, tachycardia, hypertension, hyponatraemia, mental status changes, muscle weakness, and change in urine colour to red/brown.^1^, ^3^, ^4^, ^12^ Attacks often require hospitalization and, without prompt treatment, can result in paralysis, respiratory failure, and, rarely, permanent neurologic deficits or death.^4^, ^13^, ^14^ Approximately 3% to 8% of symptomatic patients with AIP experience recurrent attacks (≥4 attacks/year).^13^, ^15^, ^16^ Some patients also experience debilitating chronic symptoms between attacks, such as pain, fatigue, and nausea.^4^, ^17^ Long‐term complications and comorbidities related to AHP can include chronic kidney disease (CKD), fixed systemic arterial hypertension, chronic neuropathy, and liver disease (including aminotransferase elevations, fibrosis, cirrhosis, and hepatocellular carcinoma).^3^, ^4^, ^5^, ^16^, ^18^, ^19^, ^20^, ^21^

Prior to the approval of givosiran, treatment options were limited, and disease management focused on avoidance of attack triggers and use of intravenous (IV) glucose or hemin for attacks.^12^ For patients experiencing recurrent attacks, the impact of the disease can be severe^4^, ^17^; management may include prophylactic hemin, and, rarely, liver transplantation has been used as the treatment of last resort.^6^, ^22^ Hemin treatment carries the risk of adverse events (AEs), both acute (eg, headache, phlebitis) and chronic (eg, iron overload, venous thrombosis, venous obliteration, and central venous catheter complications).^5^, ^10^, ^12^, ^23^

Givosiran is a subcutaneously administered RNA interference therapeutic approved for the treatment of AHP in adults (USA, Brazil, Canada),^24^, ^25^, ^26^ and in adults and adolescents aged 12 years and older (European Economic Area, United Kingdom, Switzerland, Japan).^27^ Targeting messenger RNA (mRNA) encoding ALAS1, givosiran lowers induced ALAS1, thereby preventing accumulation of ALA and PBG.^28^, ^29^, ^30^, ^31^ Clinical studies have demonstrated that givosiran treatment leads to sustained lowering of urinary ALAS1 mRNA, ALA and PBG levels, and, in patients experiencing recurrent attacks, reduces the annualized attack rate (AAR) compared with placebo.^30^, ^32^

Givosiran treatment for 6 months during the double‐blind period of the randomized, placebo‐controlled, phase 3 study in 94 patients with AHP and recurrent attacks (ENVISION) led to reductions in porphyria attack rate, hemin usage, ALA and PBG levels, and daily worst pain compared with placebo.^28^ Patients treated with givosiran also showed improvement in QOL and patient‐reported outcomes. After the double‐blind period, all on‐study patients received givosiran during the open‐label extension (OLE) period, which aims to assess the long‐term efficacy and safety of givosiran in patients with AHP. Here we report interim data from the patients in ENVISION who completed at least 24 months on study.

## 2 MATERIALS AND METHODS

### 2.1 Study design and patients

ENVISION (NCT03338816) is a 36‐month study evaluating the efficacy and safety of givosiran in patients with AHP: a 6‐month, double‐blind, randomized, placebo‐controlled period,^28^ and a 30‐month OLE period. The present analysis reflects cumulative efficacy and safety data as of the data cutoff date of June 24, 2020, at which time all active patients had at least completed the Month 24 visit. Eligible patients were aged ≥12 years with a documented diagnosis of AHP and a confirmed AHP genetic mutation or biochemical and clinical criteria consistent with AHP, had ≥2 porphyria attacks (requiring hospitalization, urgent healthcare visit, or treatment with IV hemin at home) within the 6 months prior to baseline, and agreed to discontinue prophylactic hemin (hemin only permitted for acute attacks). During the double‐blind period, patients were randomized (1:1) to monthly givosiran (2.5 mg/kg) or placebo for 6 months.

Patients entering the 30‐month OLE received subcutaneous givosiran 2.5 or 1.25 mg/kg monthly through Month 12 (Figure S1). The lower dose was introduced in a protocol amendment to assess efficacy and safety. Those enrolled before the amendment received 2.5 mg/kg; therefore, dose allocation in the OLE was not balanced. Patients receiving 1.25 mg/kg who experienced inadequate disease control could revert to 2.5 mg/kg at or after the Month 13 visit. In a further protocol amendment, all patients remaining on the lower dose with no clinically relevant transaminase elevations had their doses increased to 2.5 mg/kg. The study was approved by central and local institutional review boards or ethics committees and was conducted in accordance with Good Clinical Practice guidelines and the provisions of the Declaration of Helsinki. All patients provided written informed consent.

### 2.2 Outcome measures and safety assessments

Efficacy assessments including AAR of composite porphyria attacks (defined as attacks requiring hospitalization, urgent healthcare visit, or IV hemin administration at home and hereinafter referred to as “composite attacks” or “attacks”), annualized days of hemin use, and urinary levels of ALA and PBG were collected throughout the study. Patient‐reported outcomes included daily worst pain, fatigue, and nausea (Figure S2),^33^, ^34^ opioid use, changes from baseline in the 12‐Item Short Form Health Survey Version 2 (SF‐12v2) scores,^35^ EuroQOL‐5 Dimension (EQ‐5D), Patient Global Impression of Change (PGIC), and Porphyria Patient Experience Questionnaire (PPEQ) (Figures S3, S4, and S5). Data for daily worst pain, fatigue, nausea, opioid use, and PGIC were collected through Month 12. Safety assessments included monitoring of AEs, clinical laboratory measures, vital signs, 12‐lead electrocardiography, and physical examination and were done throughout the study. Adverse events were coded according to the *Medical Dictionary for Regulatory Activities* Version 23.0.

### 2.3 Statistical analysis

This 24‐month interim analysis was conducted using data with a cutoff date of June 24, 2020, when all active study patients had completed their Month 24 visit. Efficacy and patient‐reported outcomes were analysed according to whether patients received givosiran in the double‐blind period before receiving givosiran in the OLE (continuous givosiran group) or received placebo in the double‐blind period and crossed over to givosiran in the OLE (placebo crossover group). Analyses of efficacy outcomes were descriptive. Safety assessments were analysed in all patients who received at least one dose of givosiran; cumulative safety data from first dose of givosiran through June 24, 2020 were reported.

## 3 RESULTS

### 3.1 ENVISION population

Of 94 patients enrolled in the double‐blind period, 89 had AIP, two had VP, one had HCP, and two had AHP without identified mutations. Of the 48 patients randomized to receive givosiran in the double‐blind period, 47 entered the OLE (continuous givosiran group). One patient with VP did not enter the OLE due to abnormal liver function tests and treatment discontinuation (discussed in Safety section). All 46 patients randomized to receive placebo in the double‐blind period entered the OLE and began givosiran treatment (placebo crossover group). In the OLE, 37 patients received givosiran 1.25 mg/kg monthly (n = 20 in the continuous givosiran group and n = 17 in the placebo crossover group), and 56 patients received givosiran 2.5 mg/kg monthly (n = 27 in the continuous givosiran group and n = 29 in the placebo crossover group). Of the 37 patients who initially received 1.25 mg/kg monthly in the OLE, 18 experienced inadequate disease control (n = 9 in the continuous givosiran group and n = 9 in the placebo crossover group) and received 2.5 mg/kg at or after the Month 13 visit. All patients remaining on the lower dose received 2.5 mg/kg following a subsequent protocol amendment (approximately half of these escalations occurred after the data cutoff date of June 24, 2020) (Figure S1). Continuous givosiran and placebo crossover groups were generally well balanced with respect to baseline demographic and clinical characteristics (Table 1).

**TABLE 1:** Baseline demographic and clinical characteristics of patients with acute hepatic porphyria in the ENVISION study

Characteristic	Placebo crossover (n = 46)	Continuous givosiran (n = 48)	All givosiran (N = 94)
Age at screening, years, median (range)	36.0 (20, 60)	42.0 (19, 65)	37.5 (19, 65)
Female, n (%)	41 (89)	43 (90)	84 (89)
Race, n (%)			
Caucasian	34 (74)	39 (81)	73 (78)
Black/African American	1 (2)	0 (0)	1 (1)
Asian	7 (15)	8 (17)	15 (16)
Other	4 (9)	1 (2)	5 (5)
AIP, n (%)	43 (93)	46 (96)	89 (95)
Non‐AIP,^a^ n (%)	3 (7)	2 (4)	5 (5)
HCP	0 (0)	1 (2)	1 (1)
VP	1 (2)	1 (2)	2 (2)
AHP without an identified mutation^b^	2 (4)	0 (0)	2 (2)
Years since diagnosis, median (range)	6.46 (0.1, 38.5)	6.98 (0.2, 43.3)	6.55 (0.1, 43.3)
Prior hemin prophylaxis, n (%)	18 (39)	20 (42)	38 (40)
Historical AAR,^c^ median (range)	7.0 (0,^d^ 46)	8.0 (4, 34)	8.0 (0,^d^ 46)
Prior chronic symptoms,^e^ n (%)	26 (57)	23 (48)	49 (52)
Prior chronic opioid use,^f^ n (%)	13 (28)	14 (29)	27 (29)
Baseline urinary ALA (mmol/mol Cr), median (range)	16.4 (1.4, 41.5)	16.4 (1.8, 88.9)	16.4 (1.4, 88.9)
Baseline urinary PBG (mmol/mol Cr), median (range)	39.3 (3.6, 87.7)	39.6 (0.4, 150.0)	39.6 (0.4, 150.0)
Neuropathy, n (%)	16 (35)	20 (42)	36 (38)
Sensory	8 (17)	10 (21)	18 (19)
Motor	8 (17)	13 (27)	21 (22)
Autonomic	3 (7)	0	3 (3)

Note

AAR, annualized rate of composite porphyria attacks; AHP, acute hepatic porphyria; AIP, acute intermittent porphyria; ALA, delta‐aminolevulinic acid; Cr, creatinine; HCP, hereditary coproporphyria; IV, intravenous; OLE, open‐label extension; PBG, porphobilinogen; VP, variegate porphyria.

^a^ Porphyria subtypes other than acute intermittent porphyria include HCP, VP, ALA dehydratase–deficiency porphyria with an identified mutation, and acute hepatic porphyria without an identified mutation. No patients with ALA dehydratase–deficiency porphyria were enrolled in this trial.

^b^ The two patients with acute hepatic porphyria without an identified mutation were considered by the trial investigator to have acute intermittent porphyria on the basis of biochemical analysis.

^c^ Composite porphyria attacks are attacks requiring hospitalization, an urgent healthcare visit, or IV hemin treatment at home.

^d^ One patient in the placebo group did not meet inclusion criterion of ≥2 composite porphyria attacks within 6 months prior to screening (patient had 2 attacks that were treated at home without IV hemin). This was identified as a protocol deviation.

^e^ Symptoms were chronic if patients experienced symptoms of porphyria daily or on most days when not having an attack and were reported by Investigators.

^f^ Opioid use was defined as chronic if patients reported taking them for porphyria daily or most days when not having an attack.

At data cutoff, 10 patients had discontinued treatment and 7 patients had withdrawn from the study (Figure S1); overall, 87 givosiran‐treated patients remained in the study. Primary reasons for treatment discontinuation were AEs (n = 4, 1 in the double‐blind period and 3 in the OLE), pregnancy (n = 1), noncompliance with study drug (n = 1), and participant decision (n = 4).

As of June 24, 2020, overall median exposure to givosiran was 22.2 (range, 1.8‐30.4) months (1 month = 30.44 days), with a cumulative exposure of 164.0 person‐years. A total of 89, 87, 75, 28, and 2 patients received givosiran for ≥6, ≥12, ≥18, ≥24, and ≥30 months, respectively.

### 3.2 Efficacy

#### 3.2.1 Annualized attack rate and hemin use

**FIGURE 1:**
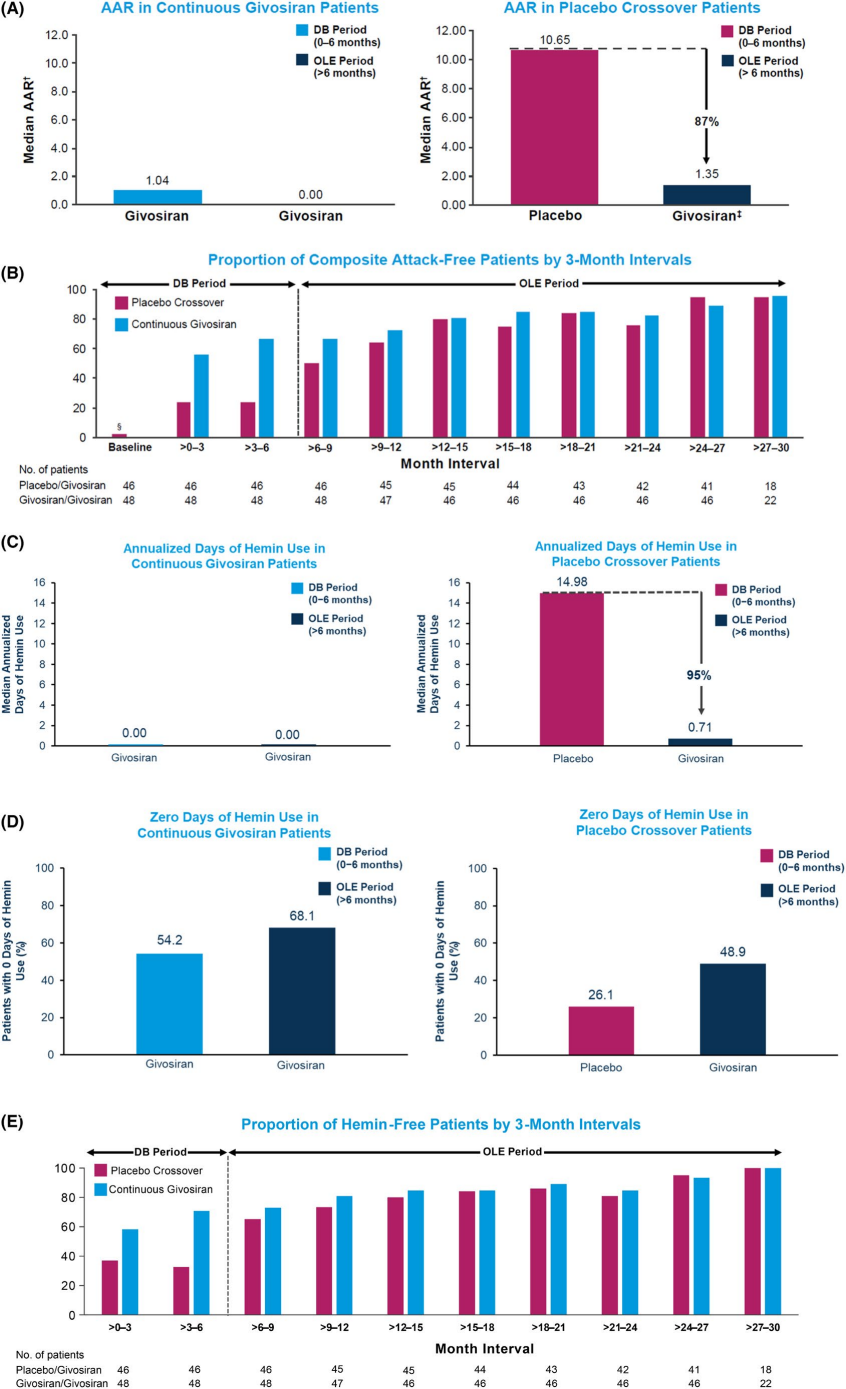
Attack frequency and hemin use with long‐term givosiran treatment. A, Median AAR. ^†^Descriptive analysis. ^‡^Placebo crossover patients receiving givosiran 2.5 mg/kg (n = 29) or 1.25 mg/kg (n = 17). B, Proportion of patients with zero attacks by 3‐month intervals. Baseline represents 6 months prior to randomization. ^§^One patient did not meet an inclusion criterion and was enrolled in the study (did not have the requisite number of attacks in the 6 months prior to randomization). Composite attacks include porphyria attacks requiring hospitalization, urgent healthcare visit, or intravenous hemin administration at home. 1 month = 28 days. C, Median annualized days of hemin use. D, Proportion of patients with zero days of hemin use. E, Proportion of patients with zero days of hemin use by 3‐month intervals. AAR, annualized attack rate; DB, double‐blind; Givo, givosiran; OLE, open‐label extension; PBO, placebo

Long‐term treatment with givosiran led to sustained AAR reduction (Figure 1A). Patients in the continuous givosiran group had a sustained AAR reduction (median AAR 1.00 and 0.00 during the double‐blind and OLE periods, respectively). In the placebo crossover group, median AAR decreased from 10.65 in the double‐blind period to 1.35 in the OLE. During givosiran treatment, median AAR was 0.46 and 1.35 in the continuous givosiran and placebo crossover groups, respectively, and 0.63 in all givosiran patients. The proportion of patients with zero composite attacks per 3‐month interval increased during the OLE compared with the double‐blind period from 67% at Month >3 to 6 to 83% at Month >21 to 24 (continuous givosiran group) and from 24% to 76% (placebo crossover group) (Figure 1B).

Long‐term givosiran treatment was associated with a sustained reduction in hemin use (Figure 1C). In the continuous givosiran group, median annualized days of hemin use were 0.00 during the double‐blind period (0‐6 months) and 0.00 during the OLE (>6 months). From baseline until the data cutoff date (double‐blind + OLE periods), overall median annualized days of hemin use across all patients treated with givosiran was 0.44 day per year. The proportion of patients with zero days of hemin use increased during the OLE compared with the double‐blind period (Figure 1D) and reached ≥93% in both the placebo crossover and continuous givosiran groups by Month 27 in the OLE period (Figure 1E). In the continuous givosiran group, 68% of patients did not require hemin during the OLE. In the placebo crossover group, 49% of patients had zero days of hemin use during the OLE compared with 26% in the double‐blind period.

#### 3.2.2 Patient‐reported outcomes and patient experience

Patients in the continuous givosiran group reported a further decrease in daily worst pain during the OLE (median changes from baseline score of 2.29 in daily worst pain were −0.34 and −0.77 in the double‐blind period and OLE period Month 6 to Month 12, respectively). Placebo crossover patients also reported a decrease in daily worst pain in the OLE compared with the double‐blind period (median changes from baseline score of 3.50 in daily worst pain were +0.10 in the double‐blind period and −0.54 in the OLE). Decreases in the number of patients (67% vs 83%) and median proportion of days (5.7 vs 8.5) with opioid use were reported in the placebo crossover group during the OLE compared with the double‐blind period. No changes were observed in patient‐reported scores for fatigue and nausea.

At Month 24, patients with long‐term treatment with givosiran showed further improvement in physical and mental health, as assessed by the SF‐12 Physical Component Summary (PCS), Mental Component Summary (MCS), and individual domain scores, all of which increased compared with Month 6 in both the continuous givosiran and placebo crossover groups (SF‐12v2 survey; Figures S6A and S6B). Givosiran treatment also further improved QOL assessed by the EuroQol‐visual analog scale element of the EQ‐5D during the 24‐month OLE (Figure S7). Patient‐rated overall status (PGIC; Figure S8) was assessed until 12 months; the majority of patients in the continuous givosiran group reported improvement in overall status since study commencement at Months 6 and 12; placebo crossover patients had similar improvements at Month 12 compared with givosiran patients at Month 6. Long‐term treatment with givosiran was associated with improvement in activities of daily living, perception of treatment, and living a more normal life, with improvements at 24 months seen in both the continuous givosiran and placebo crossover groups (PPEQ; Figures S9A and S9B).

#### 3.2.3 Pharmacodynamics

**FIGURE 2:**
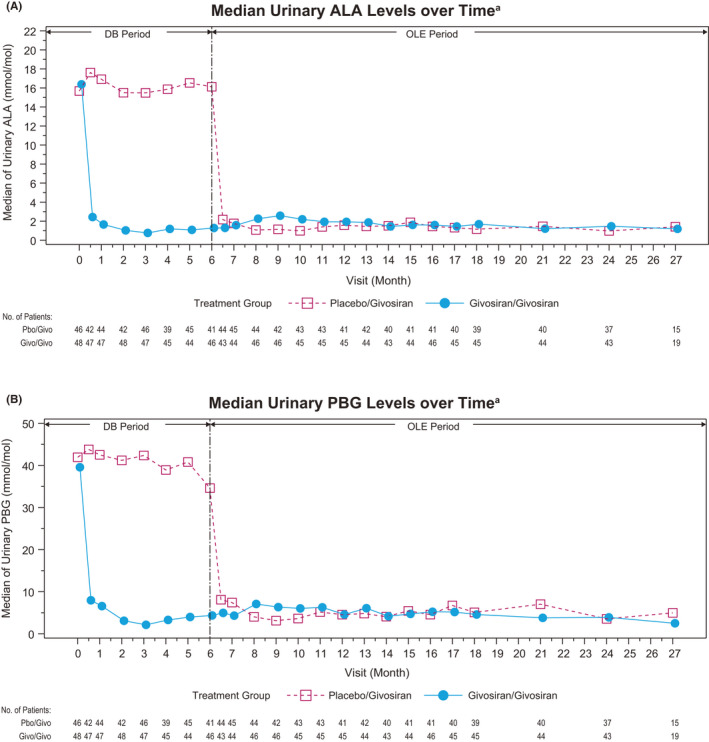
Urinary ALA and PBG levels. A, Median ALA levels over time. B, Median PBG levels over time. OLE data for 1.25 and 2.5 mg/kg are pooled. Reference ranges: ALA ULN, 1.47 mmol/mol Cr; PBG ULN, 0.14 mmol/mol Cr.^52^ ALA, delta‐aminolevulinic acid; Cr, creatinine; DB, double‐blind; OLE, open‐label extension; PBG, porphobilinogen; ULN, upper limit of normal

Long‐term givosiran treatment led to a sustained lowering of median urinary ALA and PBG to near‐normal levels in the continuous givosiran group, and a >75% reduction in the placebo crossover group during the OLE (Figures 2A and B). At baseline during the double‐blind period, mean urinary ALAS1 mRNA expression was similar in the placebo and givosiran groups (2.21 and 2.66, respectively). At Month 6, mean ALAS1 mRNA was reduced by 58% in the givosiran group and increased by 12% in the placebo group (data not shown).

### 3.3 Safety

AEs were reported in 90 (96%) patients; the majority of events were mild or moderate in severity. Severe AEs were reported in 27 (29%) patients. The most frequently reported AEs (in ≥10% of patients) were injection‐site reaction (ISR), nausea, fatigue, nasopharyngitis, and headache (Table 2).

**TABLE 2:** Safety overview in patients with AHP during givosiran treatment

n (%)	Placebo crossover (n = 46)	Continuous givosiran (n = 48)	All givosiran (N = 94)
Any AE	43 (94)	47 (98)	90 (96)
AEs occurring in ≥10% of patients			
Injection‐site reactions^a^	16 (35)	19 (40)	35 (37)
Nausea	11 (24)	21 (44)	32 (34)
Fatigue	10 (22)	12 (25)	22 (23)
Nasopharyngitis	11 (24)	11 (23)	22 (23)
Headache	7 (15)	12 (25)	19 (20)
Urinary tract infection	8 (17)	9 (19)	17 (18)
Upper respiratory tract infection	10 (22)	6 (13)	16 (17)
Vomiting	8 (17)	7 (15)	15 (16)
Diarrhoea	7 (15)	7 (15)	14 (15)
Abdominal pain	6 (13)	7 (15)	13 (14)
Lipase increased	6 (13)	6 (13)	12 (13)
Constipation	4 (9)	6 (13)	10 (11)
Influenza	5 (11)	5 (10)	10 (11)
AEs of interest			
Hepatic AEs^b^	8 (17)	9 (19)	17 (18)
Renal AEs^c^			
Any event	9 (20)	12 (25)	21 (22)
Increased serum creatinine or decreased eGFR^d^	8 (19)	13 (27)	21 (22)
Any serious AE	13 (28)	15 (31)	28 (30)
Any severe AE	14 (30)	13 (27)	27 (29)
Any AE leading to treatment discontinuation	2 (4)	1 (2)	3 (3)
Any AE leading to study withdrawal	2 (4)	1 (2)	3 (3)
Death	0	0	0

Note

Safety data from first dose of givosiran to data cutoff date (June 24, 2020).

AE, adverse event; AHP, acute hepatic porphyria; eGFR, estimated glomerular filtration rate; MedDRA, *M* *edical Dictionary for Regulatory Activities;* SMQ, standardized MedDRA query.

^a^ Injection‐site reactions include all AEs included under the term of high‐level injection‐site reactions in MedDRA.

^b^ Hepatic AEs included any AEs within the SMQ drug‐related hepatic disorders.

^c^ Renal AEs included all AEs mapping to the SMQ chronic kidney disease.

^d^ This category included a subgroup of patients who had changes in serum creatinine level or eGFR reported as an increased blood creatinine level, a decreased eGFR, or chronic kidney disease.

A total of 28 (30%) patients reported serious AEs (SAEs) during the study; SAEs reported in >1 patient were blood homocysteine increased, CKD, device breakage, pyrexia, and urinary tract infection (all n = 2) (Table S1). Three patients discontinued treatment because of SAEs considered related to givosiran by the investigators. One patient discontinued treatment because of abnormal results on liver function testing (reported during the double‐blind period and described below). Two patients at one site had SAEs of blood homocysteine increased, based on laboratory assessments performed by the investigator that were not prespecified in the protocol. The elevations of homocysteine were considered medically significant events and considered possibly related to givosiran by the investigator. One of these patients had a concurrent SAE of hypersensitivity, and the other had a concurrent SAE of pancreatitis.^36^ Both withdrew from the study due to the SAEs of blood homocysteine increased. There were no deaths related to givosiran during the study.

Hepatic AEs were reported in 17 (18%) patients; all were mild or moderate in severity, the majority being serum aminotransferase elevations. A total of 10 patients (11%) had alanine aminotransferase (ALT) levels more than 3 times the upper limit of normal (ULN), of whom 3 patients (3%) had ALT levels more than 5 times ULN. One patient with ALT greater than 8 times the ULN, reported as an SAE of liver function test abnormal, discontinued treatment (due to a protocol‐defined stopping rule) and withdrew from the study at the end of the double‐blind period. The ALT elevations generally occurred approximately 3 to 6 months after givosiran was started, and then resolved subsequently (Figure S10). No patients discontinued givosiran due to hepatic events during the OLE period.

Twenty‐one patients (22%) reported renal AEs, which were mostly increased creatinine and/or decreased estimated glomerular filtration rate (eGFR); most events were mild or moderate in severity and none led to treatment discontinuation. Small decreases in eGFR observed early in therapy stabilized over Months 12 to 24 (Figure S11). Some patients with pre‐existing kidney disease showed a small, continued decrease in their eGFR. No patients discontinued givosiran due to renal events during the OLE period.

ISRs occurred in 37% of patients and 6% of the 2152 doses of givosiran given; all ISRs were mild or moderate in severity, and none led to discontinuation. The most common symptoms of ISRs included erythema, pruritus, rash, pain, and swelling at the injection site.

On laboratory evaluation, there have been no notable changes in haematology parameters related to givosiran. Mean values for lipase and amylase have remained generally stable during the study; however, intermittent elevations of lipase and amylase have been observed in some patients. The proportion of patients with shifts in lipase and amylase were comparable between the placebo and givosiran groups during the double‐blind period, without any imbalances.

## 4 DISCUSSION

Disease burden is substantial and treatment options are limited for patients with AHP who experience recurrent attacks and chronic symptoms between attacks.^4^, ^12^, ^17^, ^37^ Natural history data suggest that up to 65% experience chronic, debilitating symptoms such as pain, fatigue, and nausea that negatively impact daily functioning and QOL.^4^, ^28^, ^38^, ^39^, ^40^ Hemin is recommended for treatment of acute attacks that do not respond to treatment with glucose and in patients who display neurologic symptoms or require hospitalization^7^, ^41^, ^42^; it is also used for prophylaxis.^4^, ^43^ However, repeated prophylactic use of hemin may be associated with reduced efficacy, and it is associated with AEs such as venous damage and thrombophlebitis, coagulation abnormalities, and secondary iron overload.^5^, ^28^, ^44^, ^45^ Compared to placebo, givosiran treatment has been shown to have significant clinical efficacy and an acceptable safety profile in patients with AHP.^28^

Consistent with the results from the double‐blind period, this 24‐month interim analysis of the ENVISION study confirms that long‐term givosiran dosing leads to continuous and sustained reductions in AAR and hemin use, with 83% and 76% of patients being attack‐free (continuous givosiran and placebo crossover groups, respectively), and 68% and 49% of patients, respectively, not requiring supplemental hemin. Lower opioid use occurred against a background of patient‐reported reduced daily worst pain (12‐month data). Long‐term givosiran dosing resulted in improvements in several patient‐reported outcomes, including physical functioning, activities of daily living, and overall health status assessment scores. The SF‐12 PCS score increased by 8.9 points in the continuous givosiran group and 10.0 points in placebo crossover patients in the OLE. In other chronic diseases, a ≥2‐ to 5‐point increase is considered a clinically meaningful improvement.^38^, ^39^ Sustained and continuous improvements in the attack rate, the proportion of patients who remained attack‐free, and patient‐reported outcomes were associated with sustained lowering of ALA and PBG levels, the toxic heme intermediates considered causal for disease manifestations.^2^, ^11^

During the OLE period, the protocol was amended to assess the efficacy and safety of a lower dose of givosiran (1.25 mg/kg). Results demonstrated a trend toward greater reductions in AAR, urinary ALA and PBG levels, and hemin use in placebo crossover patients treated with givosiran 2.5 mg/kg once monthly, compared with those treated with givosiran 1.25 mg/kg once monthly (Alnylam, data on file). Consistent with this observation, approximately half of the patients assigned to the 1.25 mg/kg dosing regimen (including those in the placebo crossover group and the continuous givosiran group) had inadequate disease control and required dose escalation to 2.5 mg/kg. Both dosing regimens had acceptable safety profiles. Thus, the recommended dosing regimen for givosiran is 2.5 mg/kg once monthly.

The key safety findings of this 24‐month interim analysis were consistent with those observed during the 6‐month double‐blind period^28^ and from the phase 2 OLE study, in which patients were treated with givosiran for ≥36 months.^46^ Elevations in serum aminotransferase levels occurred in some patients, primarily 3 to 5 months after initiation of the trial regimen; most resolved with continued dosing. CKD is a long‐term complication of AHP,^18^ and one‐third of patients in ENVISION had reduced eGFR (<60 mL/min/1.73 m^2^) at baseline. During treatment with givosiran, small (mostly reversible) decreases in eGFR were observed early in therapy and generally stabilized by Months 12 to 24. Renal function should be monitored during givosiran treatment, as clinically indicated.

Elevations of blood homocysteine have been reported in patients with AHP, with a correlation of higher levels in those with greater disease activity.^36^, ^47^, ^48^, ^49^ As 2 SAEs of blood homocysteine increases were observed in the present ENVISION study,^36^ analyses of blood homocysteine levels were performed on exploratory samples, which included levels collected before, during, and after givosiran treatment. During these analyses, blood homocysteine levels were also noted to be increased compared with available baseline levels.^50^, ^51^ Blood homocysteine levels increased in all patients in one analysis (9/9 patients) and in most patients in the second analysis (14/15 patients).^50^, ^51^ The degree of homocysteine elevation varied among patients.^50^, ^51^ The long‐term consequences of homocysteine elevations in patients with AHP are unknown. Additional work on the possible implications is needed.

The study is limited by the relatively small number of patients in the study population. However, the ongoing ENVISION study is the largest intervention study to date for this rare disease.

The 24‐month data from this phase 3 study show that long‐term dosing with givosiran is well tolerated and provides sustained and continuous benefit to patients with AHP, as reflected by a durable reduction in frequency of attacks, hemin use to treat attacks, levels of toxic heme intermediates ALA and PBG, daily pain, and opioid use. Givosiran treatment was also associated with improvement in assessments of physical functioning and QOL.

## ETHICS APPROVAL STATEMENT

The study was approved by central and local institutional review boards or ethics committees and was conducted in accordance with Good Clinical Practice guidelines and the provisions of the Declaration of Helsinki.

## CONFLICT OF INTEREST

Dr Ventura reported receiving advisory board fees and lecture fees from Alnylam Pharmaceuticals and advisory board fees from Recordati Rare Diseases. Dr Bonkovsky reported receiving grant support and financial support, paid to Wake Forest University School of Medicine, from Alnylam Pharmaceuticals, Gilead Sciences, and Mitsubishi Tanabe, NA; consulting fees from Alnylam Pharmaceuticals, Disc Medicine, Eiger Biopharmaceuticals, Protagonist Therapeutics, and Recordati Rare Diseases. Dr Gouya reported receiving travel support and financial support from Alnylam Pharmaceuticals. Dr Aguilera‐Peiró reported receiving advisory board fees from Alnylam Pharmaceuticals. Dr Bissell reported receiving financial support, paid to University of California, San Francisco, from Alnylam Pharmaceuticals. Dr Stein reported receiving consulting fees, registration reimbursement, and financial support, paid to King's College Hospital, from Alnylam Pharmaceuticals. Dr Balwani reported receiving grant support, consulting fees, advisory board fees, and lecture fees from Alnylam Pharmaceuticals, advisory board fees from Recordati Rare Diseases, grant support and advisory board fees from Mitsubishi Tanabe, and advisory board fees from Alexion, Genzyme/Sanofi, and Takeda. In addition, Mount Sinai faculty are named Co‐Inventors with Alnylam on a patent related to the development of givosiran, the study drug. The Icahn School of Medicine at Mount Sinai receives payments related to this patent from Alnylam, and a portion of these payments are also distributed to faculty and other co‐inventors. Dr Anderson reported receiving grant support and consulting fees from Alnylam Pharmaceuticals, Recordati Rare Diseases, and Mitsubishi Tanabe, and consulting fees from Moderna Therapeutics. Dr Parker reported receiving financial support, paid to University of Utah, from Alnylam Pharmaceuticals. Dr Kuter reported receiving grant support and consulting fees from Actelion (Syntimmune), Agios, Alnylam Pharmaceuticals, Amgen, Argenx, Bristol Myers Squibb, Protalix, Rigel, and Takeda (Bioverativ), grant support from Kezar and Principia, and consulting fees from Caremark, Daiichi Sankyo, Dova, Kyowa‐Kirin, Merck Sharp Dohme, Momenta, Novartis, Pfizer, Platelet Disorder Support Association, Principia, Protalix, Sanofi, Genzyme, Shionogi, Shire, UCB, Up‐To‐Date, and Zafgen. Dr Monroy reported receiving advisory board fees from Alnylam Pharmaceuticals. Dr Oh reported lecture fees from Merck, Pfizer, and Genzyme. Dr Ritchie reported receiving consulting fees, paid to the University of Alberta from Alnylam, Takeda, CSL Behring, and BioCryst, and grant support from CSL Behring and OctaPharma. Drs. Ko, Hua, and Sweetser reported being employed by and owning stock and stock options in Alnylam Pharmaceuticals. Dr Sardh reported receiving grant support and personal fees, paid to Karolinska Institutet, from Alnylam Pharmaceuticals.

## AUTHORS CONTRIBUTIONS

All authors had full access to all of the data in the study and take responsibility for the integrity of the data and the accuracy of the data analysis. Concept and design: Anderson, Balwani, Bissell, Bonkovsky, Gouya, Sardh. Acquisition, analysis, or interpretation of data: All authors. Drafting of the manuscript: Ko, Hua. Critical revision of the manuscript for important intellectual content: All authors. Statistical analysis: Hua. Supervision: Sardh, Ventura.

## ADDITIONAL CONTRIBUTIONS

Joseph Bloomer, MD (University of Alabama, USA), Daphne Vassiliou, MD, PhD (Karolinska University Hospital, Sweden), Elisabeth Minder, MD (Stadtspital Triemli, Switzerland), John Phillips, PhD (University of Utah School of Medicine, USA), David Rees, MD (King's College Hospital, UK), Ashwani Singal, MD (University of Alabama, USA) and Bruce Wang, MD (University of California, USA) also contributed to the design and concept of the study. Editorial assistance was provided by Jennifer LS Willoughby (PhD) of Alnylam Pharmaceuticals, Cambridge, MA, USA. Medical writing services were provided by Colette Szarka (MPhil) of Adelphi Communications Ltd, Macclesfield, UK, and by Michael Morren (RPh, MBA) of Peloton Advantage, LLC, an OPEN Health company, Parsippany, NJ, USA, in accordance with Good Publication Practice (GPP3) guidelines, and were funded by Alnylam Pharmaceuticals, Cambridge, MA, USA.

## TRIAL REGISTRATION NUMBER

ClinicalTrials.gov, NCT03338816.

## PATIENT CONSENT STATEMENT

All patients provided written informed consent.

## Supporting information

Supplementary Material

## Data Availability

De‐identified individual participant data that support these results will be made available in a secure‐access environment 12 months after study completion and when the product and indication have been approved for no less than 12 months in the US and the EU. Access will be provided contingent upon the approval of a research proposal and the execution of a data sharing agreement.
