# Supplementary material for: Efficacy and safety of givosiran for acute hepatic porphyria: 24‐month interim analysis of the randomized phase 3 ENVISION study
Source: Liver Int. 2021 Nov 16;42(1):161–72. doi: 10.1111/liv.15090 (PMC9299194; doi:10.1111/liv.15090)
Supplement: Supplementary file 1 — Supplementary Material [file LIV-42-161-s001.docx]

**SUPPORTING INFORMATION FOR**

Efficacy and safety of givosiran for acute hepatic porphyria:

24-month interim analysis of the randomized phase 3 ENVISION study

Paolo Ventura, Herbert L. Bonkovsky, Laurent Gouya, Paula Aguilera-Peiró,
D. Montgomery Bissell, Penelope E. Stein, Manisha Balwani, D. Karl E. Anderson, Charles Parker, David J. Kuter, Susana Monroy, Jeeyoung Oh, Bruce Ritchie,
John J. Ko, Zhaowei Hua, Marianne T. Sweetser, Eliane Sardh,
for the ENVISION Investigators

**ENVISION Collaborators**

David C. Rees, Ulrich Stölzel, Jerzy Windyga, Samuel M. Silver, Siobán B. Keel, Jiaan-Der Wang, Pauline Harper, Daphne Vassiliou, Bruce Wang, John Phillips, Aneta Ivanova, Janneke G. Langendonk, Raili Kauppinen, Elisabeth Minder, Yutaka Horie, Petro Petrides, Appalanaidu Sasapu, Gayle Ross, Ming-Jen Lee, Hung-Chou Kuo, Ole Hother-Nielsen, Bruno Sangro, Encarna Guillén Navarro, Manish Thapar, Peter Stewart, David Coman, Tomohide Adachi, Yoshie Goto, Kei-ichiro Takase, David Cassiman, Maria Cappellini, Ashwani Singal, Cynthia Levy, Fumio Nakahara, Matteo Marcacci, Bettia Celestin, and Delia D’Avola.

*Affiliations*

King’s College London, King’s College Hospital, London, UK (Rees), Klinikum Chemnitz, Chemnitz, Germany (Stölzel), Department of Hemostatic Disorders and Internal Medicine, Institute of Hematology and Transfusion Medicine, Warsaw, Poland (Windyga), University of Michigan, Ann Arbor, MI, USA (Silver), School of Medicine, Division of Hematology, University of Washington, Seattle, WA, USA (Keel), Center for Rare Disease and Hemophilia, Taichung Veterans General Hospital, Taichung, Taiwan (J-D Wang), Porphyria Centre Sweden, Centre for Inherited Metabolic Diseases, Karolinska Institutet, Karolinska University Hospital, Stockholm, Sweden (Harper, Vassiliou), UCSF Liver Center and Porphyria Center, University of California, San Francisco, CA, USA (B Wang), University of Utah, Salt Lake City, UT, USA (Phillips), St. Ivan Rilski University Hospital, Sofia, Bulgaria (Ivanova), Porphyria Center Rotterdam, Centre for Lysosomal and Metabolic Disease, Department of Internal Medicine, Erasmus MC, University Medical Center Rotterdam, The Netherlands (Langendonk), Department of Medicine, University Hospital of Helsinki, Helsinki, Finland (Kauppinen), Stadtspital Triemli, Zentrallabor, Zurich, Switzerland (Minder), Tottori University School of Medicine, Tottori, Japan (Horie), Praxis für Hämatologie und Onkologie, Isartor Zweibrückenstr, Munich, Germany (Petrides), University of Arkansas for Medical Sciences, Little Rock, AR, USA (Sasapu), Royal Melbourne Hospital, Parkville, Victoria, Australia (Ross), National Taiwan University Hospital, Taipei City, Taiwan (Lee), Chang Gung Medical Foundation, Taoyuan City, Taiwan (Kuo), Odense University Hospital, Odense, Denmark (Hother-Nielsen), Clinica Universidad de Navarra, Pamplona, Spain (Sangro), Hospital Universitario Virgen de la Arrixaca, Murcia, Spain (Guillén Navarro), Thomas Jefferson University, Philadelphia, PA, United States (Thapar), Royal Prince Alfred Hospital, Camperdown, Australia (Stewart), The Wesley Hospital, Auchenflower, Australia (Coman), Tokyo Saiseikai Central Hospital, Tokyo, Japan (Adachi), JA Shizuoka Kohseiren Enshu Hospital, Hamamatsu, Japan (Goto), Iizuka Hospital, Aso Co., Iizuka, Japan (Takase); University Hospital Leuven, Leuven, Belgium (Cassiman), University of Milan, Milan, Italy (Cappellini), University of Alabama, Birmingham, AL, USA (Singal), University of Miami, Miami, FL, USA (Levy), Hiroshima University Hospital, Hiroshima, Japan (Nakahara), Department of Surgical and Medical Sciences for Children and Adults, Internal Medicine Unit, University of Modena and Reggio Emilia, Modena, Italy (Marcacci), University of Paris, Paris, France; Centre de Référence Maladies Rares Porphyries, APHP, Colombes, France and; Laboratory of Excellence GR-Ex (Celestin), Clinica Universidad de Navarra, Madrid, Spain (D’Avola).

**ENVISION Data Monitoring Committee Members**

David E. Cohen (Chair), Janet Turks Wittes, Jules L. Dienstag, and Steven I. Shedlofsky

*Affiliations*

Cornell Weill School of Medicine, New York City, NY, USA (Cohen), Statistics Collaborative, Inc., Washing, DC, USA (Turks Wittes), Massachusetts General Hospital, Boston, MA, USA (Dienstag), University of Kentucky, Lexington, KY, USA (Shedlofsky).

**Table S1.** Serious Adverse Events in Patients with AHP During Givosiran Treatment

| **Event, n (%)** | **Placebo Crossover (n=46)** | **Continuous Givosiran (n=48)** | **All Givosiran**  **(N=94)** |
| --- | --- | --- | --- |
| Any serious adverse event^†^ | 13 (28) | 15 (31) | 28 (30) |
| Blood homocysteine increased | 2 (4) | 0 (0) | 2 (2) |
| Chronic kidney disease | 0 (0) | 2 (4) | 2 (2) |
| Device breakage | 1 (2) | 1 (2) | 2 (2) |
| Pyrexia | 1 (2) | 1 (2) | 2 (2) |
| Urinary tract infection | 1 (2) | 1 (2) | 2 (2) |
| Abdominal pain | 0 (0) | 1 (2) | 1 (1) |
| Administration site extravasation | 1 (2) | 0 (0) | 1 (1) |
| Asthenia | 1 (2) | 0 (0) | 1 (1) |
| Asthma | 0 (0) | 1 (2) | 1 (1) |
| Bronchitis | 1 (2) | 0 (0) | 1 (1) |
| Catheter bacteremia | 0 (0) | 1 (2) | 1 (1) |
| Cerebral venous sinus thrombosis | 0 (0) | 1 (2) | 1 (1) |
| Cholelithiasis | 0 (0) | 1 (2) | 1 (1) |
| *Clostridium difficile* infection | 1 (2) | 0 (0) | 1 (1) |
| Colon neoplasm | 1 (2) | 0 (0) | 1 (1) |
| Device-related infection | 0 (0) | 1 (2) | 1 (1) |
| Drug dependence | 0 (0) | 1 (2) | 1 (1) |
| Drug withdrawal syndrome | 1 (2) | 0 (0) | 1 (1) |
| Duodenal ulcer | 0 (0) | 1 (2) | 1 (1) |
| Edema | 1 (2) | 0 (0) | 1 (1) |
| Electrolyte imbalance | 0 (0) | 1 (2) | 1 (1) |
| Gastritis | 1 (2) | 0 (0) | 1 (1) |
| Gastroenteritis | 0 (0) | 1 (2) | 1 (1) |
| Headache | 0 (0) | 1 (2) | 1 (1) |
| Helicobacter gastritis | 0 (0) | 1 (2) | 1 (1) |
| Hiatus hernia | 1 (2) | 0 (0) | 1 (1) |
| Hypoglycemia | 0 (0) | 1 (2) | 1 (1) |
| Hysterosalpingo-oophorectomy | 0 (0) | 1 (2) | 1 (1) |
| Influenza | 0 (0) | 1 (2) | 1 (1) |
| Injection site reaction | 1 (2) | 0 (0) | 1 (1) |
| Invasive breast carcinoma | 1 (2) | 0 (0) | 1 (1) |
| Invasive ductal breast carcinoma | 1 (2) | 0 (0) | 1 (1) |
| Kidney infection | 1 (2) | 0 (0) | 1 (1) |
| Liver function test abnormal | 0 (0) | 1 (2) | 1 (1) |
| Lower respiratory tract infection | 0 (0) | 1 (2) | 1 (1) |
| Lymphadenitis | 0 (0) | 1 (2) | 1 (1) |
| Major depression | 0 (0) | 1 (2) | 1 (1) |
| Migraine with aura | 0 (0) | 1 (2) | 1 (1) |
| Nausea | 1 (2) | 0 (0) | 1 (1) |
| Nephropathy | 1 (2) | 0 (0) | 1 (1) |
| Obstructive pancreatitis | 1 (2) | 0 (0) | 1 (1) |
| Pain management | 0 (0) | 1 (2) | 1 (1) |
| Pancreatitis | 1 (2) | 0 (0) | 1 (1) |
| Panic attack | 1 (2) | 0 (0) | 1 (1) |
| Phlebitis | 1 (2) | 0 (0) | 1 (1) |
| Pulmonary embolism | 0 (0) | 1 (2) | 1 (1) |
| Pyelonephritis | 0 (0) | 1 (2) | 1 (1) |
| Septic shock | 1 (2) | 0 (0) | 1 (1) |
| Subclavian vein occlusion | 0 (0) | 1 (2) | 1 (1) |
| Subclavian vein thrombosis | 1 (2) | 0 (0) | 1 (1) |
| Transaminases increased | 1 (2) | 0 (0) | 1 (1) |
| Tremor | 1 (2) | 0 (0) | 1 (1) |
| Trismus | 1 (2) | 0 (0) | 1 (1) |
| Viral infection | 0 (0) | 1 (2) | 1 (1) |
| Vomiting | 1 (2) | 0 (0) | 1 (1) |

AHP, acute hepatic porphyria.

^†^ Three serious adverse events were reported as related to study drug: chronic kidney disease, abnormal liver function test, and transaminase increased.

**Figure S1.** Patient Disposition in the ENVISION OLE at Data Cutoff

^†^Patients assigned to 1.25 mg/kg who experienced inadequate disease control (n=18, 9 in the givosiran/givosiran groups and 9 in the placebo/givosiran groups) were allowed to have their monthly dose increased to 2.5 mg/kg starting at the Month 13 study visit. In addition, following a protocol amendment subsequent to the data cutoff for this report, all patients on 1.25 mg/kg dose with no clinically relevant transaminase elevations had their dose increased to 2.5 mg/kg monthly (7 patients received 1–2 doses of givosiran at 2.5 mg/kg prior to the data cutoff date of June 24, 2020 for the present analysis; the rest had their dose escalated to 2.5 mg/kg afterward). DB, double-blind; OLE, open-label extension; QM, once monthly.


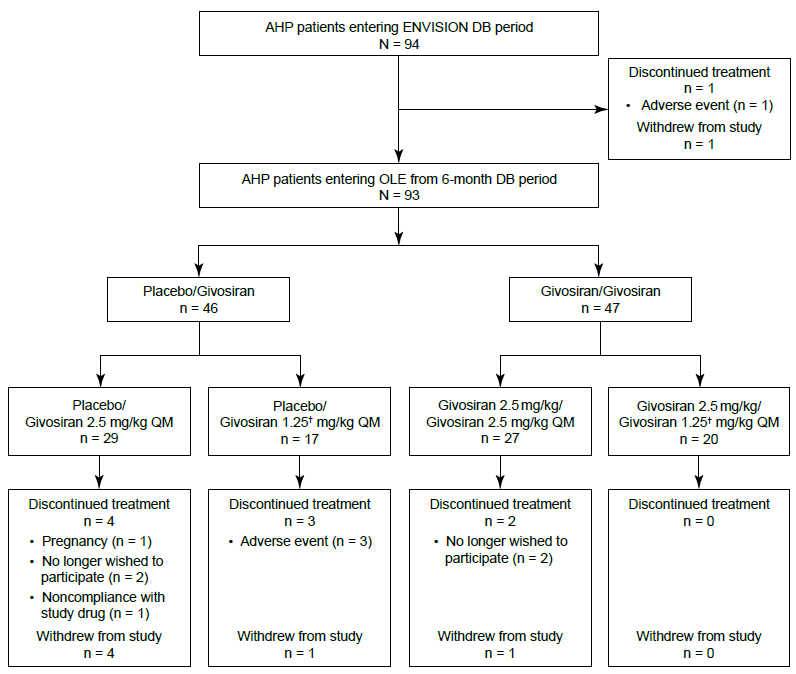


**Figure S2.** Daily Worst Pain, Fatigue, and Nausea were Documented by Study Participants in an e-diary

Patient-reported outcomes were selected following a review of the literature and the results of qualitative research conducted with AHP patients. The numeric rating scales measure the impact of the cardinal AHP symptom of pain as well as secondary AHP symptoms of fatigue and nausea. AHP, acute hepatic porphyria.


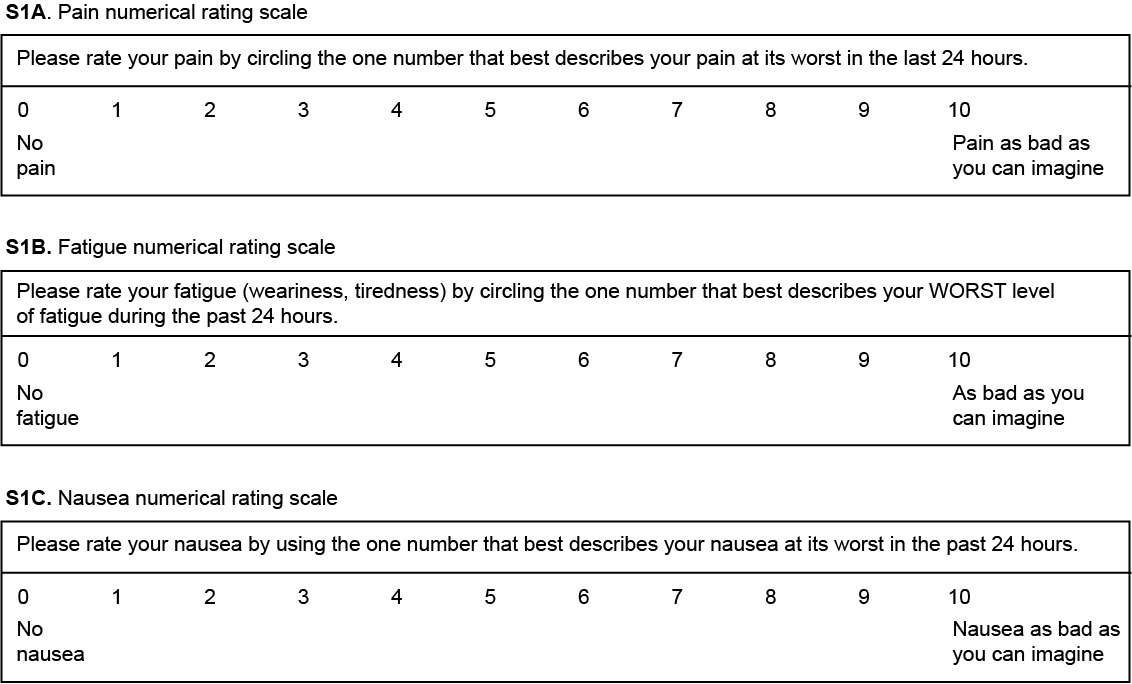


**Figure S3.** Short Form 12 Health Survey (SF-12v2) Items, Subscales, and Component Summary Measures

The SF-12 is a self-reported health-related quality of life survey comprising a Physical Component Summary (PCS), a Mental Component Summary (MCS), and eight individual health domains. EVGFP, Excellent, Very Good, Good, Fair, Poor.


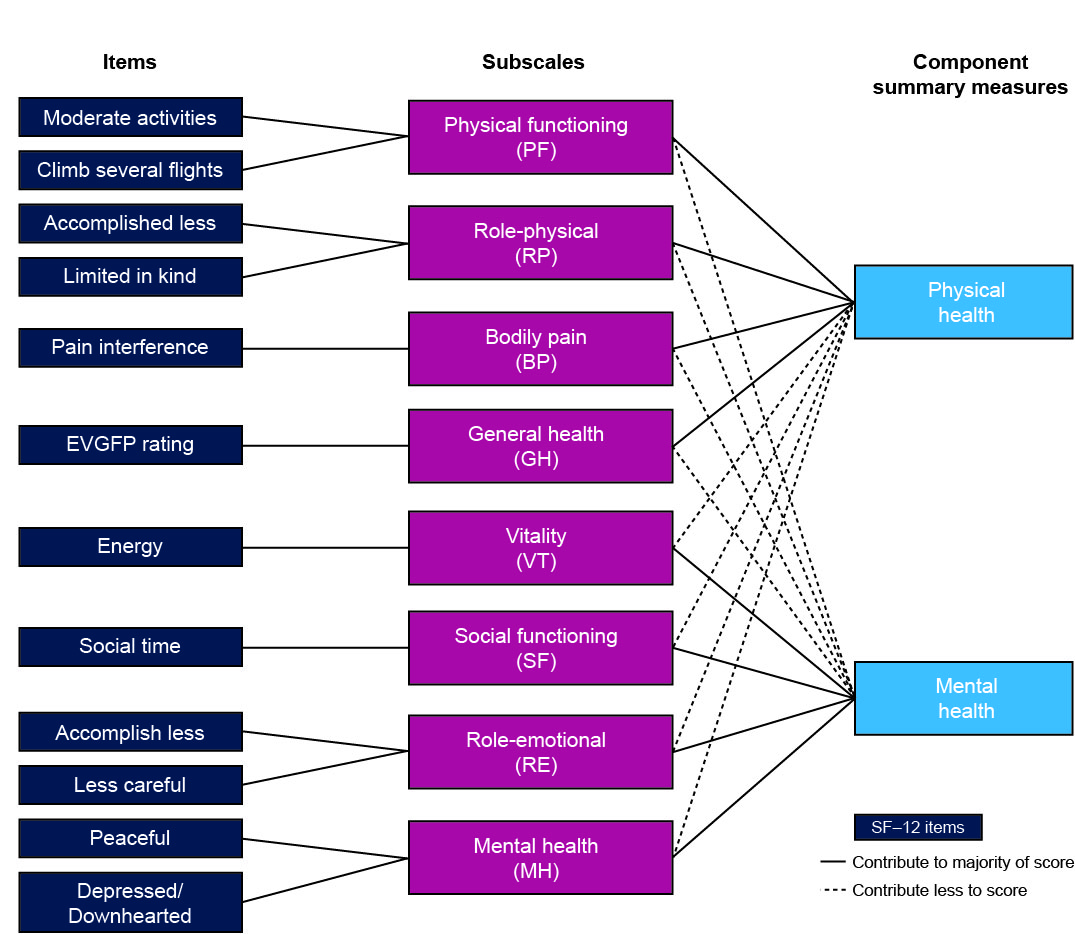


**Figure S4.** Patient Global Impression of Change

The Patient Global Impression of Change (PGIC) is a single-item, global, patient-reported outcome measure that assesses the change (improvement or worsening) in overall status relative to the start of the study. The PGIC is a global rating of change scale with no baseline assessment; patients are asked to recall changes since study commencement.


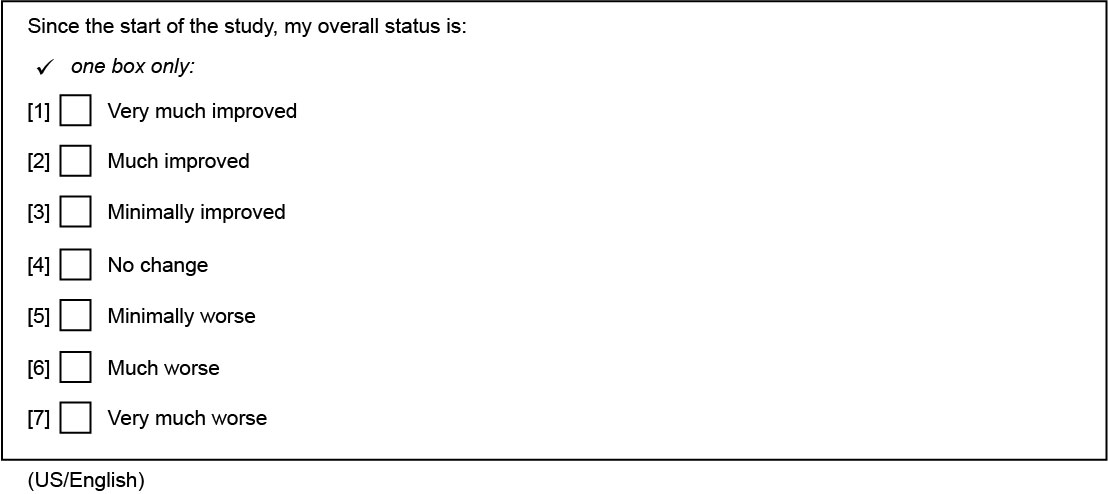


**Figure S5.** Porphyria Patient Experience Questionnaire

The Porphyria Patient Experience Questionnaire (PPEQ) assesses activities of daily living, perception of treatment, and extent to which the study drug has helped patients return to a more normal life. This custom instrument was designed to include aspects of quality of life and treatment that are important to patients but not included in other endpoints. PPEQ is a global rating of change scale with no baseline assessment; patients are asked to recall changes since study commencement.


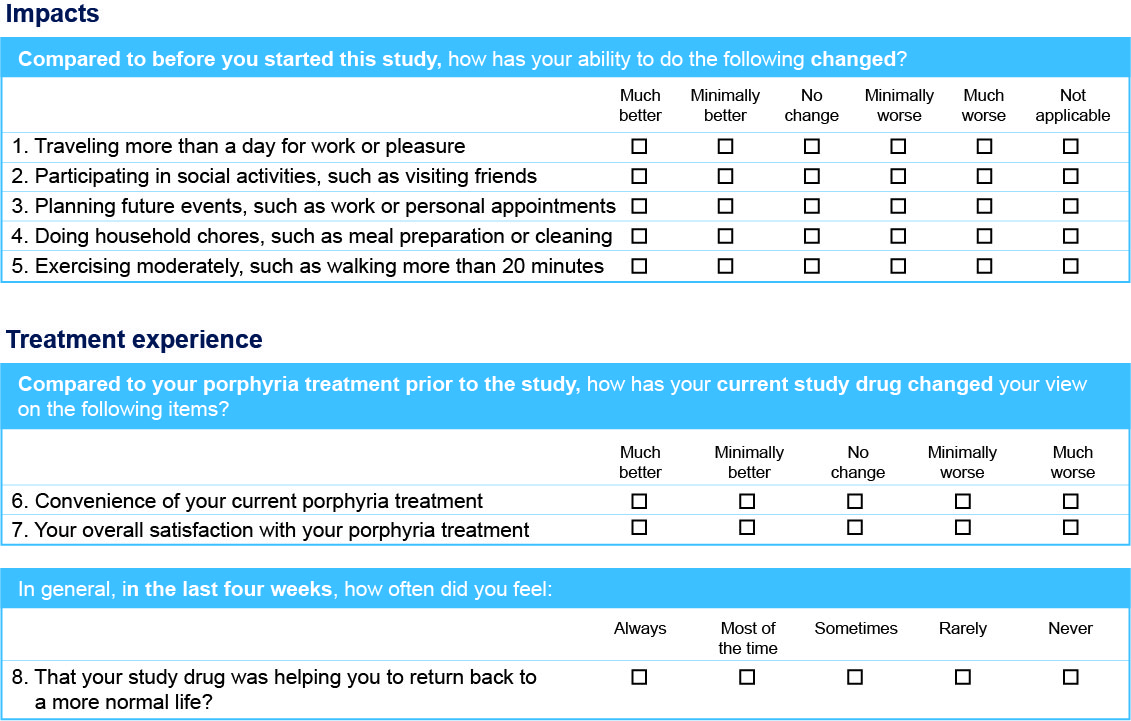


**Figure S6.** Mean Change from Baseline in PCS, MCS, and All Domains of the SF-12v2 Survey

A, Continuous givosiran. B, Placebo crossover. Higher scores represent an improvement in that summary or domain. DB, double-blind; MCS, Mental Component Summary; OLE, open-label extension; PCS, Physical Component Summary; SF-12, Short Form-12 Health Survey.

**Figure S7.** Mean Change from Baseline in EQ-VAS Score in the Continuous Givosiran and Placebo Crossover Groups

DB, double-blind; EQ-VAS, EuroQol-visual analog scale; Givo, givosiran; OLE, open-label extension; Pbo, placebo.

**Figure S8.** Patients Reporting Overall Status on the Patient Global Impression of Change Measure in the Continuous Givosiran and Placebo Crossover Groups

DB, double-blind; OLE, open-label extension.


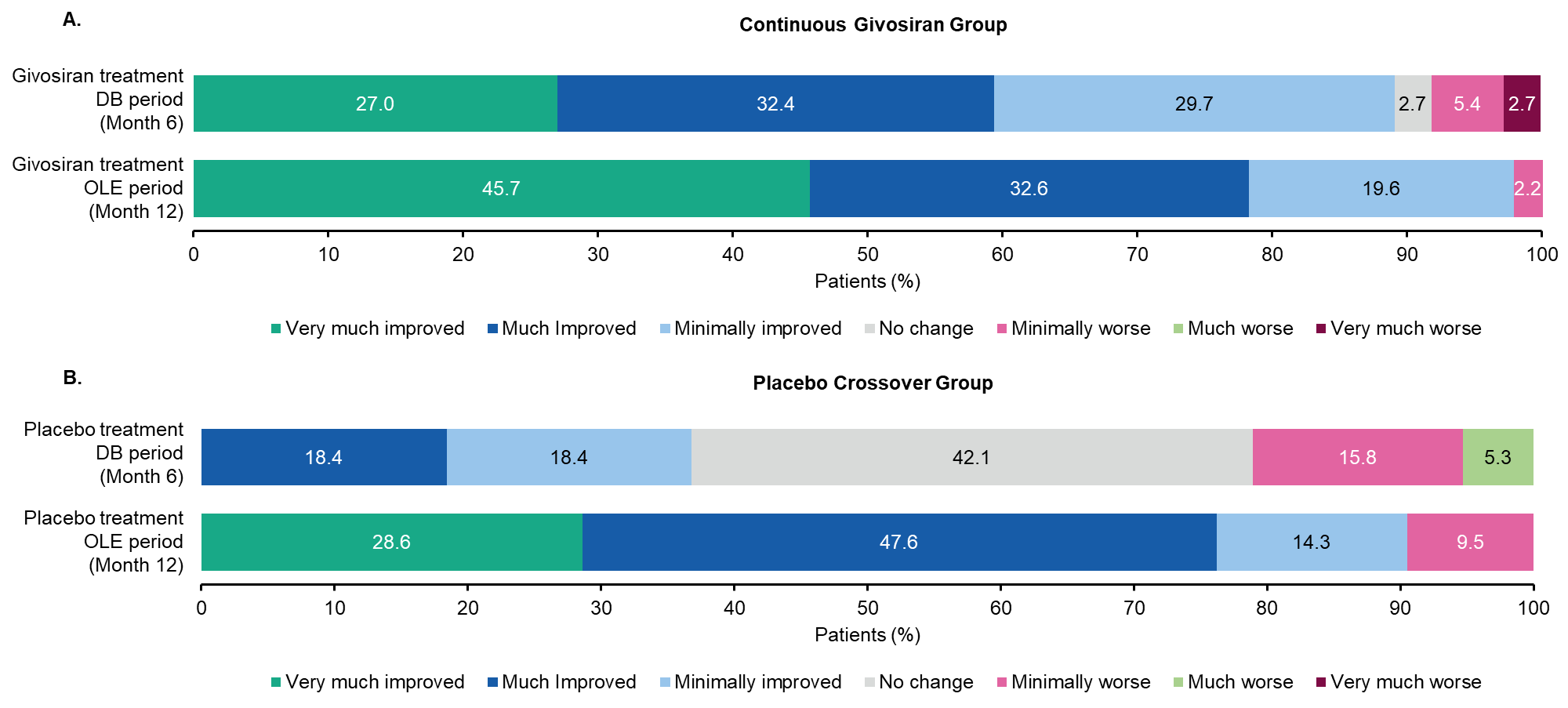


**Figure S9.** Percentage of Patients with Responses of “Much Better” or “Always” in Porphyria Patient Experience Questionnaire Categories

A, Continuous givosiran. B, Placebo crossover. Higher scores represent an improvement in that category. DB, double-blind; OLE, open-label extension; PPEQ, Porphyria Patient Experience Questionnaire.

**Figure S10.** Alanine Aminotransferase Relative to ULN During Treatment with Givosiran

ALT, alanine aminotransferase; Givo, givosiran; ULN, upper limit of normal.

**Figure S11.** Estimated Glomerular Filtration Rate During Treatment with Givosiran

eGFR, estimated glomerular filtration rate; Givo, givosiran.
